# Platelet-rich plasma attenuates the UPEC-induced cystitis via inhibiting MMP-2,9 activities and downregulation of NGF and VEGF in Canis Lupus Familiaris model

**DOI:** 10.1038/s41598-024-63760-y

**Published:** 2024-06-13

**Authors:** Ahmed I. Abdelgalil, Aya M. Yassin, Marwa S. Khattab, Elshymaa A. Abdelnaby, Sherif A. Marouf, Haithem A. Farghali, Ibrahim A. Emam

**Affiliations:** 1 Department of Surgery, Anesthesiology, and Radiology, Faculty of Veterinary Medicine, Cairo University, Giza, 12211 Egypt; 2 Department of Biochemistry and Molecular Biology, Faculty of Veterinary Medicine, Cairo University, Giza, 12211 Egypt; 3 Department of Pathology, Faculty of Veterinary Medicine, Cairo University, Giza, 12211 Egypt; 4 Theriogenology Department, Faculty of Veterinary Medicine, Cairo University, Giza, 12211 Egypt; 5 Department of Microbiology, Faculty of Veterinary Medicine, Cairo University, Giza, 12211 Egypt

**Keywords:** Canine, Cystitis, Platelet-rich plasma, Gene expression, Oxidative stress, Doppler, Biochemistry, Cell biology, Genetics, Microbiology, Molecular biology, Physiology, Diseases, Health care, Urology

## Abstract

One of the most prevalent disorders of the urinary system is urinary tract infection, which is mostly brought on by uropathogenic *Escherichia coli* (UPEC). The objective of this study was to evaluate the regenerative therapeutic and antibacterial efficacy of PRP for induced bacterial cystitis in dogs in comparison to conventional antibiotics. 25 healthy male mongrel dogs were divided into 5 groups (n = 5). Control negative group that received neither induced infection nor treatments. 20 dogs were randomized into 4 groups after two weeks of induction of UPEC cystitis into; Group 1 (control positive; G1) received weekly intravesicular instillation of sodium chloride 0.9%. Group 2 (syst/PRP; G2), treated with both systemic intramuscular antibiotic and weekly intravesicular instillation of PRP; Group 3 (PRP; G3), treated with weekly intravesicular instillation of PRP, and Group 4 (syst; G4) treated with an intramuscular systemic antibiotic. Animals were subjected to weekly clinical, ultrasonographic evaluation, urinary microbiological analysis, and redox status biomarkers estimation. Urinary matrix metalloproteinases (MMP-2, MMP-9) and urinary gene expression for platelet-derived growth factor -B (PDGF-B), nerve growth factor (NGF), and vascular endothelial growth factor (VEGF) were measured. At the end of the study, dogs were euthanized, and the bladder tissues were examined macroscopically, histologically, and immunohistochemically for NF-κB P65 and Cox-2. The PRP-treated group showed significant improvement for all the clinical, Doppler parameters, and the urinary redox status (*p* < 0.05). The urinary MMPs activity was significantly decreased in the PRP-treated group and the expression level of urinary NGF and VEGF were downregulated while PDGFB was significantly upregulated (*p* < 0.05). Meanwhile, the urinary viable cell count was significantly reduced in all treatments (*P* < 0.05). Gross examination of bladder tissue showed marked improvement for the PRP-treated group, expressed in the histopathological findings. Immunohistochemical analysis revealed a marked increase in Cox-2 and NF-κB P65 in the PRP-treated group (*P* < 0.05). autologous CaCl2-activated PRP was able to overcome the bacterial infection, generating an inflammatory environment to overcome the old one and initiate tissue healing. Hence, PRP is a promising alternative therapeutic for UPEC cystitis instead of conventional antibiotics.

## Introduction

Urinary tract infection (UTI) is one of the most common disorders of the urinary tract, affecting both sexes of all ages^1^. Over the last 30 years, the prevalence of UTI infections increased by 60.4%, having a substantial influence on people's health, access to healthcare, and financial resources. UTI rates have been observed to range from 3 to 14% in companion animals, such as dogs and cats, and are routinely detected in veterinary care^2–7^. Most patients with UTIs have uropathogenic *E. coli* (UPEC) as their main pathogen. It causes anomalies in the urothelial barrier, releases cytokines and interleukins from the urothelium, triggers an immunological reaction, and warns the immune system of impending danger^8–10^. Many pathways have been implicated in the progression of cystitis. It has been found that elevated amounts of neurotrophins in the urine and tissues, such as brain-derived neurotrophic factor (BDNF) and nerve growth factor (NGF), in the urine and tissues. This implies that a higher incidence of urination and painful bladder could be caused by sensory nerve activation and the plasticity of neurons^11,12^, and they are therefore regarded as a crucial component in the relationship between inflammation and changed pain signaling pathways^13^. The inducible early response gene cyclooxygenase-2 (Cox-2) is triggered by several inflammatory stimuli, such as arachidonic acid, lipopolysaccharide (LPS), platelet-activating factor (PAF), tumor necrosis factor (TNF), interleukin-1 (IL-1), endothelin, and platelet-activating factor (PAF)^14–16^. This results in an overproduction of prostanoids. Cox-2 overexpression has been linked to cystitis and was seen in hemorrhagic cystitis^17^. Moreover, NF-KB can be activated by cytokines, growth factors, infections, and other agents, which will cause its activated subunits to translocate to the nucleus^18,19^. NF-KB plays a pivotal role in the chronic inflammatory process and was found to be activated in the cells of the urothelium and submucosal layer in biopsies of interstitial cystitis patients^20^. NF-κB was also an essential factor in the transcription and activation of the COX-2 gene^21^.

In cell culture, matrix metalloproteinases (MMPs) are produced in response to bacterial byproducts like lipopolysaccharide and phospholipase C^22–24^. MMPs have a direct impact on extracellular matrix (ECM) proteins, which is why they are involved in physiological tissue remodeling. Thus, it has been shown that pathological conditions like human carcinoma and inflammatory diseases have elevated MMP expression levels^25^. Regarding MMP production and mechanistics during bacterial infections, not much is known^26^. Urinary antioxidant enzyme consumption during a UTI may contribute to oxidative stress, and this protective effect may be insufficient^27^.

In cystitis, increasing perfusion in the wall of the urinary bladder is expected; therefore, Doppler ultrasonography can measure the quantity of vascularization^28–30^. UTI clinical manifestations vary from mild self-limiting to severe sepsis^31^. Although antibiotics are the first line of conventional treatment for urinary tract infections (UTIs), the emergence of bacterial resistance is significantly reducing their efficacy^32,33^. More than 80% of *E. coli* isolates from urinary tract infections (UTIs) in developing nations exhibit resistance to trimethoprim-sulfamethoxazole, ciprofloxacin, and amoxicillin-clavulanic acid^34^. As a result, the Infectious Disease Society of America (IDSA) and the World Health Organization (WHO) stated that new medications must be developed to overcome antibiotic resistance^35,36^.

An autologous combination of plasma proteins and leukocyte-free plasma is known as platelet-rich plasma (PRP). Growth factors and cytokines, such as transforming growth factor-β (TGF-β), epidermal growth factor (EGF), basic fibroblast growth factor (bFGF), insulin-like growth factor 1 (IGF-1), and platelet-derived growth factor (PDGF), can be released by platelets^37^. Research on animals has indicated that PRP administration has the potential to decrease infections caused by *Staphylococcus aureus* or gram-negative bacteria, such as *E. coli*^38,39^. Platelet-rich gel's antimicrobial qualities can help treat infections that take longer to heal. In addition, PRP injection improved the expression of barrier proteins, cytoskeleton, and urothelial cell proliferation in recurrent UTIs^40^. There is a dearth of information regarding PRP's application in managing bladder problems. Intravesical PRP injections administered repeatedly are an effective treatment for recurrent UTIs (rUTIs)^41^ and refractive interstitial cystitis (IC)^13^.

As far as the authors are aware, there is a dearth of unbiased data demonstrating the effectiveness of intravesical PRP therapy when compared to traditional techniques. To treat experimentally induced *E. Coli* cystitis in dogs, we examined the efficacy of intravesical PRP instillation both alone and in combination with systemic antibiotics in terms of clinical, ultrasonographic, biochemical, genetic, histological, and immunohistochemical evaluation.

## Methods

All methods were carried out with relevant guidelines; ARRIVE guidelines and American Veterinary Medical Association (AVMA) guidelines for the Euthanasia of Animals.

### Ethics statement

The current study's experimental protocol was given ethical approval by the Cairo University Institutional Animal Care and Use Committee (CU-IACUC) under the number (CU ӀӀ F 6 23).

### Animal model preparation

Twenty-five male mongrel dogs (14–18 kg) aged 1–3 years purchased from the Al-Fahad Trading Company of Animals (Abu-Rawash, Giza, Egypt) were clinically healthy based on the results of physical examination, ultrasonographic evaluation, CBC, serum biochemical analyses, urinalysis, and bacterial culture of a urine sample. Five dogs were used in the experiment as a control negative group for macroscopical and histopathological evaluation while the remaining 20 dogs were submitted for induced cystitis. Induced cystitis was done under the tranquilization effect of xylazine Hcl (Xylaject®, Adwia, Egypt) with a dose of 1 mg/kg. A 10 Fr folly catheter was inserted into the urethral opening and 10 ml of a 0.1% solution of alcoholic salicylic acid was infused into the urinary bladder, left inside for 20 min, and then the solution was released in the reverse direction through the catheter^2^. After 24 h, the bladder was infused with a fully identified virulent local *E. coli* strain obtained from the Department of Microbiology, Faculty of Veterinary Medicine, Cairo University, Giza, Egypt. The infective dose of *E. coli* was matched with McFarland tube 1 (3 × 10^8^ CFU/ml).

#### Study design

After 2 weeks of E.coli injection, twenty dogs were randomized by persons not aware of the study design into four groups (n = 5/group). Group 1 (control positive; G1) received weekly intravesical instillation of sodium chloride 0.9%. Group 2 (syst/PRP; G2), was treated with both systemic antibiotic and weekly intravesical instillation with PRP; Group 3 (PRP; G3), that treated with weekly intravesical instillation with PRP and Group 4 (syst; G4) which treated with a daily systemic antibiotic for 2 weeks.

#### Selection of specific antibiotic

Urine samples were collected after 12 days of induction for culture and sensitivity to select the antibiotic of choice for treatment in both Group 2 (syst/PRP; G2) and Group 4 (syst; G4). Ceftriaxone was the antibiotic of choice in this study at dose (50mg/ kg) once daily for 2 weeks..

#### Autologous PRP preparation

Whole-blood samples were taken from the jugular vein of each dog on sodium citrate tubes and were prepared using the double-spin method and activated by CaCl2 shortly before use according to^42^. Briefly, 9 ml of whole blood was collected on 1ml of 3.8% sodium citrate solution containing tubes. The tubes were then subjected to soft spin at 250xg for 10 min. The resulted upper and middle layers were collected for the following hard spin at 2000xg for 10 min. The upper 2/3 portion was removed then 1.5 ml PRP was obtained. Activation of PRP was done using 20 Mm CaCl_2_.

#### Intravesical instillation technique

Under the tranquilization effect of xylazine Hcl (Xylaject®, Adwia, Egypt) with a dose of 1 mg/kg. The dog was restrained in lateral recumbency and A 10 Fr folly catheter was inserted into the urethral opening. The catheter was advanced until reached the bladder, the urine was aspirated and then the treatment solution was infused. The medication was left inside the urinary bladder for 20 min and then the catheter was removed.

Clinical, doppler ultrasonographic, urinary biochemical, and molecular analysis and bacterial culture of a urine sample were done weekly throughout the study. Treatment was started after 2 weeks of induction with *E. coli* and extended for 2 weeks.

Euthanasia was done at the end of the study using thiopental sodium (Pharcopental 500 mg®, pharco, Egypt) at a dose of 150 mg/kg^43^ and following the AVMA Guidelines for the Euthanasia of Animals. The urinary bladder was examined macroscopically and urinary bladder sections were taken for histopathological and immune histochemical evaluations.

#### Clinical evaluation

All dogs were daily evaluated for body temperature, appetite, the color of urine, and the signs of tenesmus, dysuria, and polyuria till the end of the study.

#### B mode and color Doppler ultrasonographic evaluation

**Figure 1:**
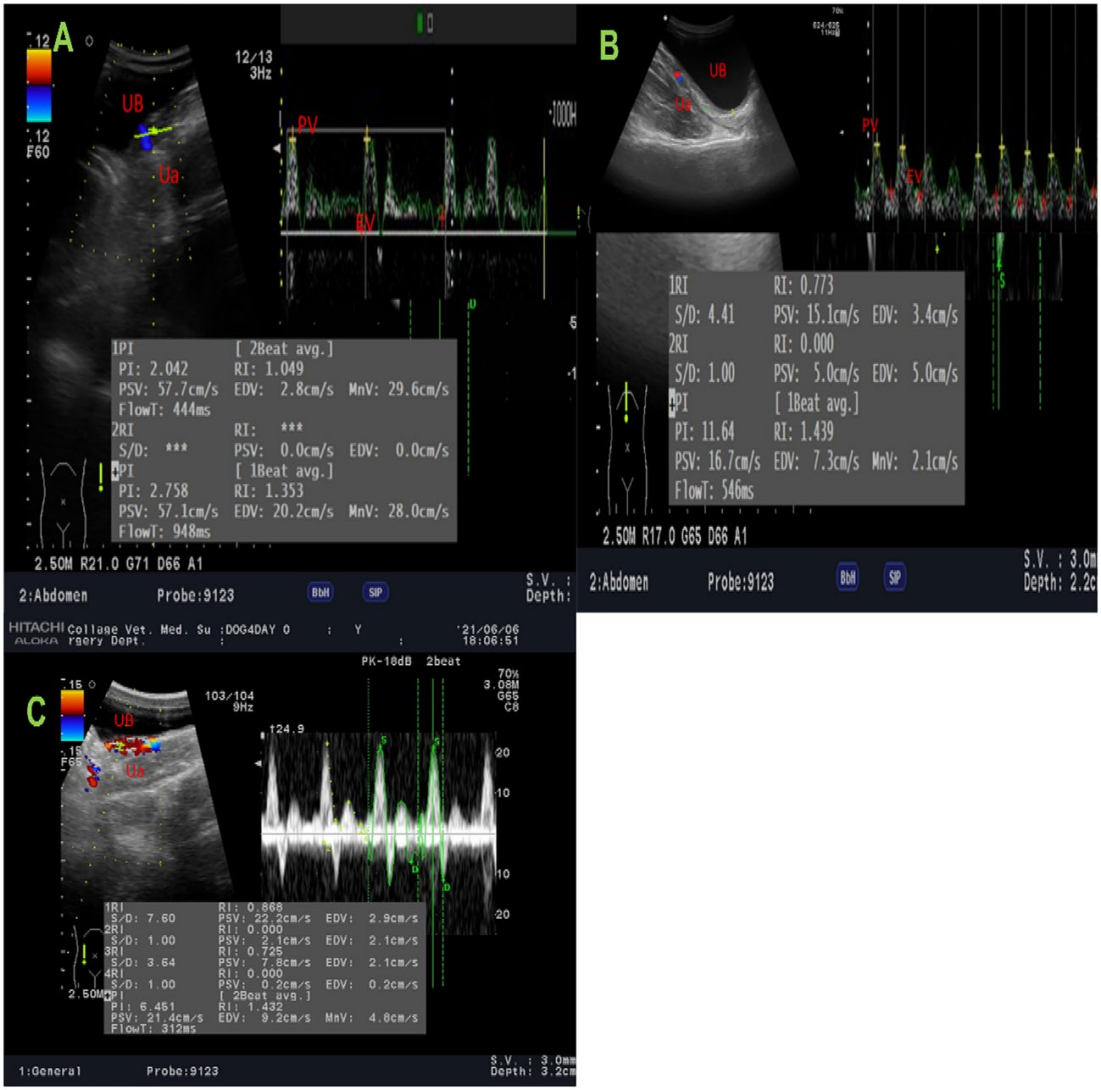
Colour and pulsed wave Doppler modes of the urinary bladder (UB) with urethral artery (Ua) blood flow waveform pattern expressed by peak and end velocities (PV and EV) to measure both Doppler indices in dogs of G 3(PRP group) suffered from cystitis. (**A**) at zero-day before treatment. (**B**) after one week of treatment. (**C**) after two weeks of treatment.

The Hitachi device Aloka F37 with Array from 5 to 12 MHz frequency and Color Doppler Velocity was used. The examination was carried out with a slow scan of the entire bladder by B-mode then color mode-activated gaining 15-s videos for later images^44,45^. Bladder thickness, urethral arterial parameters, and thickness were measured. Doppler parameters were calculated automatically as follows: pulsatility index (PI), resistance index, Doppler velocity measures as urethral peak velocity (PV) of contraction (in centimeter per second), and urethral end velocity of relaxation (EV; in centimeter per second). Device settings are maximum velocity (25 cm/s) with Doppler filter (100 Hz), PRF (4000 kHz), angle (45°), and two-color map (red + blue) (Fig. 1).

#### Urine samples preparation

Urine samples were collected from the animals under the tranquilization effect of xylazine Hcl (Xylaject®, Adwia, Egypt) with a dose of 1 mg/kg, dogs were restrained in lateral recumbency and A 10 Fr folly catheter was inserted into the urethral opening. The catheter was advanced until reached the bladder and the urine was aspirated using sterile syringe at the start of the experiment (baseline); zero-day samples; which represent the control negative samples and then weekly for 4 weeks after *E. coli* injection till the end of the study. For gene expression and zymography analysis, the samples were centrifuged at 4 °C for 15 min at 4000 × g. Until analyzed, the supernatants were stored at – 80 °C.

#### Microbiological evaluation

Urine samples were collected for microbiological analysis, viable bacterial count, total coliform count^46^, and *E. coli* isolation on Eosin Methylene blue (EMB) medium^47^ were done at days zero (before injection) and four times with week intervals (two times before treatment and two after treatment).

#### Biochemical evaluation

##### Determination of the urinary redox status

Assessment of urinary total antioxidant capacity (TAC) (mM/L)

Total antioxidant capacity (TAC) concentration (mM/L) in urine was determined using commercial kits (Bio Diagnostic, Giza, Egypt) according to the method described by according to^48^ at 505 nm.

#### Assessment of urinary malondialdehyde (MDA)

Malondialdehyde (MDA) concentration (nM/ml) was used as the index of lipid peroxidation as described by^49^. MDA was determined by measuring the thiobarbituric acid reactive species. The absorbance of the resultant pink product was measured at 534 nm.

#### Assessment of urinary nitrite

Nitrite (the stable metabolite of NO) concentration (µmol/L) was measured colorimetrically according to^50^ using commercial kits (Bio Diagnostic, Giza, Egypt) and following the manufacturer’s instructions at 540 nm.

#### Assessment of matrix metalloproteinases (MMPs) enzymes activity by gelatin zymography

A method described by^51^ was used to measure the total protein concentrations for urine samples. Gelatin zymography was used to detect the activity of MMPs (gelatinases; MMP2, and MMP9) using a technique outlined by^52^. Urine samples were separated using non-reducing SDS/PAGE on 7.5% (w/v) gels containing 1 mg/ml gelatin. The gel was left in a development buffer (0.05 M Tris/HCl, pH 8.8, 5 mM CaCl2, and 0.02% NaN3) for an entire night after being rinsed twice for fifteen minutes each time with 2.5% (v/v) Triton X-100. After that, gels were fixed and stained with 0.1% Coomassie Brilliant Blue R250 diluted with a 4.5:1:4.5 v/v/v solution of methanol, acetic acid, and water. The zymogram gels were scanned in color and then converted to grayscale for analysis using my Thermo Scientific TM Image Analysis Software.

#### Urinary gene expression analysis

##### Total RNA extraction and cDNA synthesis

Total RNA was isolated using a total RNA purification kit (Jena Bioscience, Germany, Cat. #PP-210S) according to the manufacturer’s instructions. The RNA concentration and purity were ascertained using Nanodrop ND-1000 Spectrophotometer (Thermo Scientific). The total RNA was then reversely transcribed using the Revert Aid First Strand cDNA Synthesis Kit (Thermo Scientific, USA, Cat. #K1622).

#### Real-time qPCR and gene expression analysis

The primer sequences for the vascular endothelial growth factor (VEGF), platelet-derived growth factor -B (PDGF-B), and nerve growth factor (NGF) genes were designed using Primer 3 software and illustrated in (Table 1). The mRNA expression level of each gene was determined relative to GAPDH as an endogenous reference gene through a fluorescence-based real-time detection method using iQ SYBR® Green Supermix (Bio-Rad 1,708,880, USA) according to manufacturer instructions. The cycle threshold (Ct) values were obtained using the Bio-Rad iCycler thermal cycler and the MyiQ real-time PCR detection system. The cycling protocol was set as follows: 95 °C for 3 min (initial denaturation) and then 40 cycles of denaturation at 95 °C for 15 s, annealing at 60 °C for 30 s, and extending at 72 °C for 30 s. Each assay was performed in triplicates and no-template negative control (NTC) was included; the expression relative to control was calculated using the Equation 2-ΔΔCT^53^.

**Table 1:** Primer sequences for targeted and normalizer genes.

Genes	Forward primer	Reverse primer	Product size	REF./accession No
GAPDH	5-AGGTCGGAGTCAACGGATTT-3	5-ATCTCGCTCCTGGAAGATGG-3	230	Farghali et al.^39^
VEGF	5-TCTGACTAGGAGTTCGGGGA-3	5- CCCTTCCTCCACCAATGTCT-3	214	Farghali et al.^39^
PDGF-B	5-TCGATCCGCTCCTTTGATG-3	5- GATAAACTCTCCAGCTCGTCTC-3	122	NM_001003383
NGF	5-CTGGGAGAGGTGAACATTAACA-3	5-GTAGGAGTTCCAGTGCTTGG-3	123	NM_001194950.1

#### Histopathological evaluation

Urinary bladder specimens were fixed in 10% neutral buffered formalin. Tissues were then processed in ascending concentrations of alcohol and xylene, embedded in paraffin, and sectioned by microtome (Leica RM2125, Germany) into 3 µm thick. Sections were stained with hematoxylin and Eosin stain and Masson’s trichrome. Olympus BX43 light microscope equipped with an Olympus DP27 camera was used for examination^54^.

Lesions of the urinary bladder including hyperplasia of the urothelium, leukocyte infiltration, and interstitial edema were scored according to their severity from 1 to 3 where score (1) indicates mild leukocyte infiltration in the lamina propria, mild interstitial edema, and absence or focal epithelial hyperplasia in the luminal epithelium; score (2) indicate moderate leukocytes infiltration in the lamina propria, moderate interstitial edema, and moderate generalized epithelial; and score (3) indicate severe diffuse leukocytes infiltration in the lamina propria, severe interstitial edema, and severe generalized epithelial hyperplasia^55^.

#### Immunohistochemistry

After deparaffinization of tissue sections, antigen retrieval was performed in citrate buffer PH 6. Primary antibodies either against NF-κβ P65 (sc-8008, Santa Cruz Biotechnology, Inc., Santa Cruz, CA, USA) or COX2/ Cyclooxygenase 2 monoclonal antibody (Clone SP21, Thermo Scientific) were applied overnight. Endogenous peroxidase was deactivated by the application of hydrogen peroxide. After washing, a secondary horseradish peroxidase (HRP)-labeled antibody was then applied (Universal PolyHRP DAB kit for mouse and rabbit, Genemed, Sakura Torrance, CA, USA). The translocation NF-κβ P65 expression in the nucleus was considered positive. Primary antibodies were not applied in negative control slides. Image analysis of the area percent of cox-2 and NF-κβ P65 was performed using Image J software for four captured images per animal at 200 × magnification.

#### Statistical analysis

All data obtained except the immunohistochemical results were evaluated using SPSS software 20 using analysis of variance with repeated measures followed by a post hoc analysis where appropriate. The effect of the days before and after PRP injection was calculated. In addition, Duncan’s multiple range test was employed to differentiate between significant means at *P* < 0.05. The obtained values are presented as mean ± the standard error (S.E) of the mean.

Considering immunohistochemical evaluation; all data collected were statistically analyzed using SPSS® version 20 software. Means are compared by one-way ANOVA (*P* < 0.05) according to^56^. The obtained values are presented as means ± S.E of the mean.

### Ethical approval

This work was approved by IACUC with No. (CU ӀӀ F 6 23). All methods were carried out with relevant guidelines; ARRIVE guidelines and American Veterinary Medical Association (AVMA) guidelines for the Euthanasia of Animals.

## Results

### Clinical evaluation

Decreased appetite with increased temperature (39.5 ± 2 °C) was seen after induction in four dogs. Fluid therapy was given twice daily for 4 days and then the appetite gradually improved. The body temperature became normal (38.7 ± 3 °C) after one week. Hematuria was seen for 3 days after induction and then, the color of urine became dark yellow for one week in all dogs. Dysuria and tenesmus were marked for 17 days after induction and then the signs improved in all groups except the control positive one (G1). Polyuria was typically seen in all groups all over the experiment except G3 which received intra vesicular PRP only and showed improvement after 10 days from the beginning of treatment.

### B-mode and Doppler ultrasound assessment

The urinary bladder wall thickness (mm) and the cross-sectional diameter of the urethral artery (Ua; mm) were significantly(*P* < 0.05) elevated in all experimental groups all over the studied time points compared to zero-day assessment with respect to the 2nd week post-infection which showed the highest levels of the thickness and arterial diameter for all groups. For the action of different treatments protocols; G3 demonstrated a significant decline in the thickness and arterial diameter at the 1st and 2nd weeks post-treatment (7.12 ± 0.05 mm and 7.02 ± 0.07 mm for bladder thickness; 4.95 ± 0.03mm and4.82 ± 0.07mm for the diameter of Ua), respectively, however after treatment G2 and G4 did not perform any significant difference (*P* > 0.05), while a significant difference was observed between both groups (G2 and G4) and G3 with (G1) control positive animals (*P* < 0.05; Table 2 and Table 3).

**Table 2:** Urinary Bladder (UB) wall thickness (mm) in dogs at the different time intervals for the control and treated subgroups.

Groups	Zero-day	Before treatment		After treatment	
		1st week	2nd week	1st week	2nd week
G1	10.05 ± 0.01^b^	10.62 ± 0.02^b^	11.32 ± 0.32^a^	11.55 ± 0.32^a A^	11.98 ± 0.36^a A^
G2	10.13 ± 0.55^ab^	10.42 ± 0.64^a^	10.56 ± 0.58^a^	9.12 ± 0.21^bB^	9.04 ± 0.05^b B^
G3	10.11 ± 0.25^ab^	10.66 ± 0.33^a^	10.89 ± 0.36^a^	7.12 ± 0.05^bC^	7.02 ± 0.07^bC^
G4	10.13 ± 1.21^ab^	10.26 ± 1.07^a^	10.62 ± 0.87^a^	9.22 ± 0.02^bB^	9.08 ± 0.05^bB^

Different small letter superscript values are significantly different at *P* < 0.05 in each group at different time points in the same row, while different capital letter superscript values are significantly different at *P* < 0.05 between groups at the same time point in the same column. Data represented as mean value ± standard error (S.E.) where (n = 5/ group). G1: Control positive group; G2: PRP and systemic antibiotic group; G3: PRP group, and G4: Systemic antibiotic group.

**Table 3:** Urethral artery (Ua) cross-sectional diameter (mm)in dogs at the different time intervals for the control and treated subgroups.

Groups	Zero-day	Before treatment		After treatment	
		1st week	2nd week	1st week	2nd week
G1	7.01 ± 0.99^b^	7.22 ± 0.54^b^	7.59 ± 0.05^a^	8.16 ± 0.08^a A^	8.39 ± 0.01^a A^
G2	7.03 ± 0.74^ab^	7.36 ± 0.64^a^	7.45 ± 0.32^a^	5.22 ± 0.11^b B^	5.12 ± 0.21^b B^
G3	6.85 ± 0.05^ab^	7.33 ± 0.05^a^	7.61 ± 0.01^a^	4.95 ± 0.03^b C^	4.82 ± 0.07^b C^
G4	7.01 ± 0.35^ab^	7.23 ± 0.55^a^	7.42 ± 0.21^a^	5.33 ± 0.05^b B^	5.15 ± 0.01^b B^

Different small letter superscript values are significantly different at *P* < 0.05 in each group at different time points in the same row, while different capital letter superscript values are significantly different at *P* < 0.05 between groups at the same time point in the same column. Data represented as mean value ± standard error (S.E.) where (n = 5/ group). G1: Control positive group; G2: PRP and systemic antibiotic group; G3: PRP group, and G4: Systemic antibiotic group.

Ua Doppler indices expressed by pulsatility and resistance indices (PI and RI) were significantly(*P* < 0.05) decreased in all experimental groups all over the studied time points compared to zero-day assessment with respect to the 2nd-week post-infection which showed the highest levels of the indices for all groups. For the action of different treatments protocols; G3 demonstrated a significant elevation in both indices at the 1st and 2nd weeks post-treatment 05 ± 0.01 and 2.15 ± 0.01 for Ua PI; 0.81 ± 0.01 and 0.85 ± 0.01 for Ua RI)), respectively, however after treatment G2 and G4 did not perform any significant difference (*P* > 0.05), wh ile a significant difference was observed between both groups (G2 and G4) and G3 with (G1) control positive animals(*P* < 0.05; Table 4 and Table 5).

**Table 4:** Urethral artery Doppler indices in the form of pulsatility index (PI) in dogs at the different time intervals for the control and treated subgroups.

Groups	Zero-day	Before treatment		After treatment	
		1^st^ week	2^nd^ week	1^st^ week	2^nd^ week
G1	1.86 ± 0.01^a^	1.85 ± 0.01^a^	1.81 ± 0.01^ab^	1.71 ± 0.02^b C^	1.68 ± 0.02^b C^
G2	1.86 ± 0.01^ab^	1.83 ± 0.01^b^	1.78 ± 0.01^b^	1.99 ± 0.01^a B^	2.11 ± 0.01^a B^
G3	1.87 ± 0.01^ab^	1.82 ± 0.01^b^	1.76 ± 0.01^b^	2.05 ± 0.01^a A^	2.15 ± 0.01^a A^
G4	1.88 ± 0.01^ab^	1.79 ± 0.01^b^	1.78 ± 0.01^b^	1.99 ± 0.01^a B^	2.03 ± 0.01^a B^

Different small letter superscript values are significantly different at *P* < 0.05 in each group at different time points in the same row, while different capital letter superscript values are significantly different at *P* < 0.05 between groups at the same time point in the same column. Data represented as mean value ± standard error (S.E.) where (n = 5/ group). G1: Control positive group; G2: PRP and systemic antibiotic group; G3: PRP group, and G4: Systemic antibiotic group.

**Table 5:** Urethral artery Doppler indices in the form of resistance index (RI) in dogs at the different time intervals for the control and treated subgroups.

Groups	Zero-day	Before treatment		After treatment	
		1st week	2nd week	1st week	2nd week
G1	0.68 ± 0.01^a^	0.66 ± 0.01^a^	0.58 ± 0.01^ab^	0.51 ± 0.01^bC^	0.48 ± 0.01^b C^
G2	0.67 ± 0.01^ab^	0.62 ± 0.01^b^	0.58 ± 0.01^b^	0.71 ± 0.01^aB^	0.72 ± 0.01^a B^
G3	0.66 ± 0.01^ab^	0.60 ± 0.01^b^	0.58 ± 0.01^b^	0.81 ± 0.01^a A^	0.85 ± 0.01^a A^
G4	0.68 ± 0.02^ab^	0.57 ± 0.02^b^	0.57 ± 0.02^b^	0.71 ± 0.01^a B^	0.73 ± 0.01^a B^

Different small letter superscript values are significantly different at *P* < 0.05 in each group at different time points in the same row, while different capital letter superscript values are significantly different at *P* < 0.05 between groups at the same time point in the same column. Data represented as mean value ± standard error (S.E.) where (n = 5/ group). G1: Control positive group; G2: PRP and systemic antibiotic group; G3: PRP, and G4: Systemic antibiotic group.

Ua peak point of velocity( Ua PV; cm/sec) was significantly(*P* < 0.05) increased in all experimental groups all over the studied time points compared to zero-day assessment with respect to the 2nd-week post-infection which showed the highest levels of the thickness and arterial diameter for all groups. For the action of different treatment protocols; G3 demonstrated a significant decline in the Ua PV at the 1st and 2nd weeks post-treatment (17.01 ± 0.53 cm/sec and 16.33 ± 0.99 cm/sec), respectively, however after treatment G2 and G4 did not perform any significant difference (*P* > 0.05), while a significant difference was observed between both groups (G2 and G4) and G3 with (G1) control positive animals (*P* < 0.05; Table 6), but the arterial end velocity point (EV: cm/sec) did not show any difference between groups and within the control (*P* > 0.05; Table 7) at different time points before and after different treatments protocols.

**Table 6:** Urethral artery Doppler peak velocity (PV; cm/sec) in dogs at the different time intervals for the control and treated subgroups.

Groups	Zero-day	Before treatment		After treatment	
		1st week	2nd week	1st week	2nd week
G1	21.33 ± 0.22^b^	21.58 ± 0.77^b^	21.88 ± 0.01^a^	21.89 ± 0.21^a A^	22.07 ± 0.11^a A^
G2	21.22 ± 0.82^a^	21.66 ± 0.25^a^	21.99 ± 0.74^a^	18.21 ± 0.55^b B^	18.01 ± 0.21^b B^
G3	21.34 ± 0.04^ab^	21.74 ± 0.77^a^	21.84 ± 0.95^a^	17.01 ± 0.53^b C^	16.33 ± 0.99^b C^
G4	21.29 ± 0.41^ab^	21.75 ± 0.22^a^	21.89 ± 0.25^a^	18.33 ± 0.41^b B^	18.21 ± 0.11^b B^

Different small letter superscript values are significantly different at *P* < 0.05 in each group at different time points in the same row, while different capital letter superscript values are significantly different at *P* < 0.05 between groups at the same time point in the same column. Data represented as mean value ± standard error (S.E.) where (n = 5/ group). G1: Control positive group; G2: PRP and systemic antibiotic group; G3: PRP group, and G4: Systemic antibiotic group.

**Table 7:** Urethral artery Doppler end velocity (EV; cm/sec) in dogs at the different time intervals for the control and treated subgroups.

Groups	Zero-day	Before treatment		After treatment	
		1st week	2nd week	1st week	2nd week
G1	2.76 ± 0.01	2.77 ± 0.01	2.76 ± 0.01	2.76 ± 0.01	2.75 ± 0.02
G2	2.77 ± 0.01	2.75 ± 0.01	2.75 ± 0.01	2.76 ± 0.01	2.74 ± 0.01
G3	2.75 ± 0.01	2.75 ± 0.01	2.76 ± 0.01	2.74 ± 0.01	2.75 ± 0.02
G4	2.77 ± 0.01	2.75 ± 0.01	2.77 ± 0.01	2.75 ± 0.01	2.75 ± 0.02

Different small letter superscript values are significantly different at *P* < 0.05 in each group at different time points in the same row, while different capital letter superscript values are significantly different at *P* < 0.05 between groups at the same time point in the same column. Data represented as mean value ± standard error (S.E.) where (n = 5/ group). G1: Control positive group; G2: PRP and systemic antibiotic group; G3: PRP group, and G4: Systemic antibiotic group.

### Microbiological evaluation

The microbiological profile in different groups using viable cell count (CFU/ml) was significantly increased by weeks before treatment (Table 8). After treatment, all groups significantly reduced the log count of bacteria in comparison with G1; G2 revealed a more significant group in reduction of the log count of bacteria as mean value ± standard error (S.E.) of 3.8 ± 0.05. *E. coli* detection onto the EMB medium was diminished only in G2 after 2nd week of treatment.

**Table 8:** Log10 Viable cell count (CFU/ml) in dogs in both control and experimental subgroups before and after treatment.

Groups	Zero-day	Before treatment		After treatment	
		1st week	2nd week	1st week	2nd week
G1	4.32 ± 0.047e	6.58 ± 0.37d	7.72 ± 0.31c	9.11 ± 0.25bA	10.34 ± 0.31aA
G4	4.39 ± 0.17d	5.89 ± 0.09c	8.03 ± 0.18a	6.59 ± 0.24bB	5.79 ± 0.12cB
G3	4.43 ± 0.08c	6.44 ± 0.57b	7.99 ± 0.39a	6.42 ± 0.12bB	5.32 ± 0.1cB
G2	4.63 ± 0.13d	7.07 ± 0.31b	8.07 ± 0.26a	5.34 ± 0.12cC	3.8 ± 0.05eC

^a,b^ values are significantly different at *P* < 0.05 compared with the control and experimental group with the time in the same row, while ^A, B^ values are significantly different at *P* < 0.05 between groups at the same time point in the same column. Data represented as mean value ± standard error (S.E.) where (n = 5/group). G1: Control positive group; G2: PRP and systemic antibiotic; G3: PRP; G4: Systemic antibiotic.

### Biochemical evaluation

#### Evaluation of the urinary antioxidant status

**Figure 2:**
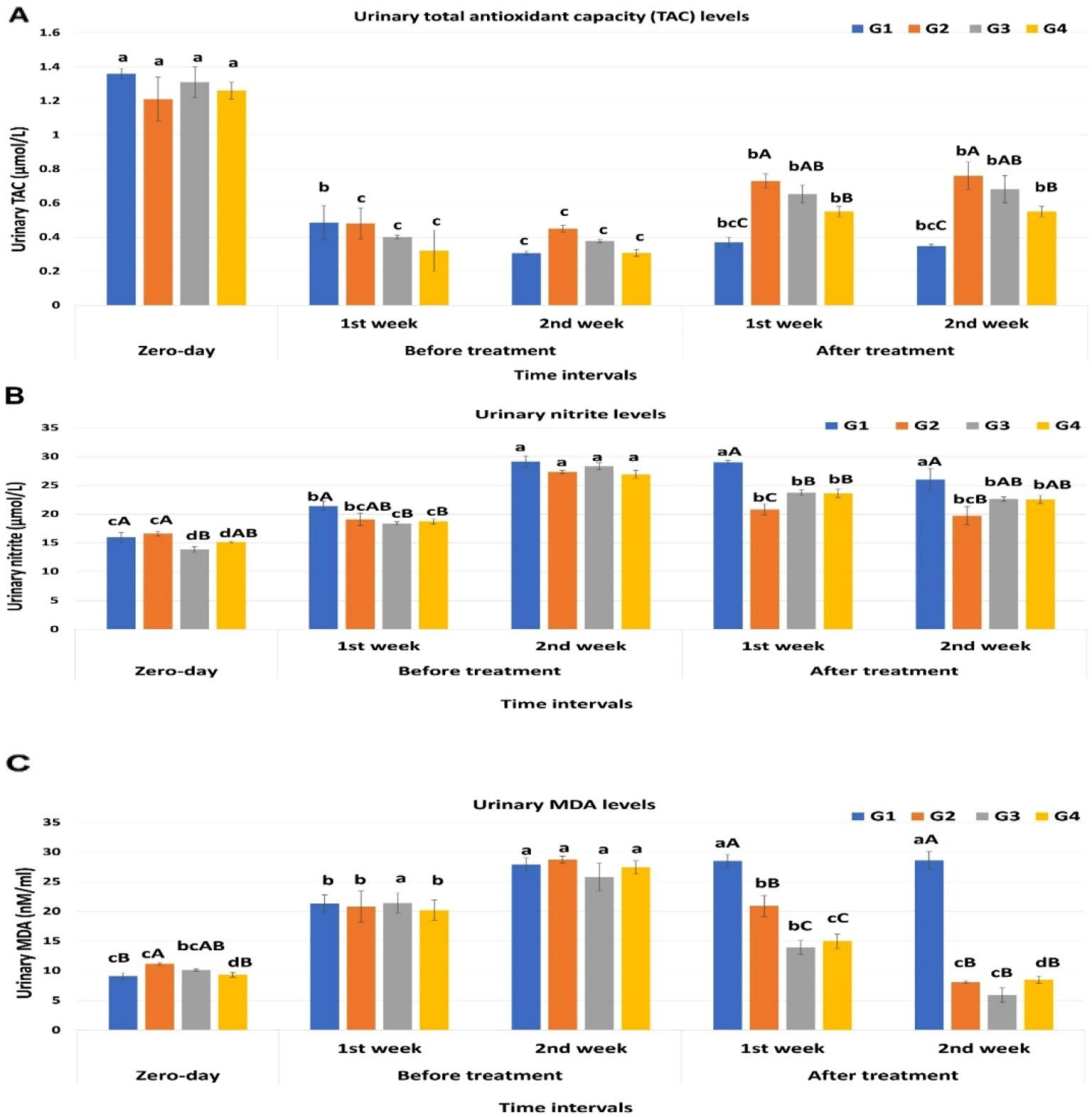
Graph representing the urinary redox status-related metabolite in dogs at different time intervals. (**A**) Urinary total antioxidant capacity (TAC) (µmol/L). (**B**) Urinary nitrite (µmol/L) levels. (**C**) Urinary MDA (nM/ml) levels. ^a,b^values are significantly different at *P* < 0.05 compared with the zero-day samples for the same group at different time intervals, while ^A,B^value is significantly different at *P* < 0.05 between groups at the same time point. Data represented as mean value ± standard error (S.E.) where (n = 5/ group). G1: Control positive group; G2: PRP and systemic antibiotic; G3: PRP; G4: Systemic antibiotic.

Urinary total antioxidant capacity (TAC) concentration significantly decreased during the 1st and 2nd week of infection in all groups with no significant difference between the groups. By the beginning of the treatment, TAC significantly increased to the periods before treatment, but it was still significantly lower than that of zero-day samples in all groups except for G1. G2 and G3 showed significantly higher concentrations than the rest of the groups in the 1st and 2nd weeks post-treatment (*P* < 0.05), Fig. 2A.

### Evaluation of urinary oxidant status

#### Urinary nitrite levels

As shown in Fig. 2B, the concentration of urinary nitrite was significantly increased in all experimental groups along the study period in comparison to the zero-day samples with regard to the 2nd week post-infection which recorded the highest concentration for all groups (*P* < 0.05). For the effect of different treatment strategies; G2 showed a significantly lower concentration between all groups for the 1st and 2nd week post-treatment 20.79 ± 0.96, 19.7 ± 1.6; respectively (*P* < 0.05), however by the end of the experiment there was no significant difference between G2, G3, and G4 19.7 ± 1.6, 22.6 ± 0.4, 22.5 ± 0.7; respectively as well as no significant difference between G1 (26 ± 1.9), G3 and G4 (*P* > 0.05).

### Urinary MDA levels

In the present study, urinary MDA showed a marked significant increase during the progression of *E. coli* infection in the 1st and 2nd week post-infection compared to the zero-time (baseline) samples (*p* < 0.05). After treatment, the MDA levels in all treated groups tended to significantly decrease to reach the baseline level at the beginning of the experiment in comparison to the untreated group while the control positive group showed a significant elevation of MDA levels. There was no significant difference between the treated groups by the end of the experiment Fig. 2C.

### Gene expression analysis

#### Urinary Platelet-derived growth factor -B gene (u PDGFB)

The relative mRNA expression level for urinary PDGF-B is illustrated in (Table 9). PDGF-B showed no significant difference between the groups as well as the different time points for each group from the zero-day samples till the 2nd-week post-infection (*P* > 0.05). Regarding the effect of the different treatments, PDGF-B was significantly upregulated at the 1st and 2nd weeks post-treatment for G2, G3, and G4. By the end of the experiment, it was upregulated by 13.1,29.2, and 7 folds for G2, G3, and G4; respectively where the significant highest increase was noticed for G3. Meanwhile, PDGF-B was significantly down-regulated at G1 by 0.43 folds at the end of the experiment (*P* < 0.05).

**Table 9:** The relative mRNA expression level of urinary PDGF-B (u PDGF-B) at the different time intervals for the control and treated subgroups.

Groups	Zero-day	Before treatment		After treatment	
		1st week	2nd week	1st week	2nd week
G1	1 ± 0.0a	0.84 ± 0.1ab	0.97 ± 0.3ab	0.5 ± 0.05abC	0.43 ± 0.14bD
G2	1 ± 0.0 c	1 ± 0.1 c	0.82 ± 0.04c	4.2 ± 0.78bB	13.1 ± 0.37aB
G3	1 ± 0.0c	0.67 ± 0.1c	0.67 ± 0.08c	6.1 ± 0.16 bA	29.2 ± 0.5aA
G4	1 ± 0.0c	0.84 ± 0.08bc	0.99 ± 0.1c	3.6 ± 0.58bB	7 ± 0.57aC

^a,b^values are significantly different at *P* < 0.05 compared with the control and experimental group with the time in the same row, while ^A,B^ values are significantly different at *P* < 0.05 between groups at the same time point in the same column. Data represented as mean value ± standard error (S.E.) where (n = 5/group). G1: Control positive group; G2: PRP and systemic antibiotic; G3: PRP; G4: Systemic antibiotic.

### Urinary growth biomarkers (u VEGF and u NGF) genes

The fold change for the u VEGF and u NGF genes are illustrated in (Tables 10 and 11); respectively. The expression level of u VEGF and u NGF was significantly upregulated during the 2 weeks of infection in comparison to the zero-day samples (*P* < 0.05) while there is no significant between the groups at these time points. During the periods of treatment, their expressions remained up-regulated till the end of the experiment for G1; meanwhile, for G2 and G4, it began to decrease in comparison to the times of infection, but it is still upregulated in comparison to zero-day samples. G3 is significantly down-regulated by 0.77 and 0.69 folds for VEGF and NGF; respectively at the end of the experiment (*P* < 0.05) with no significant difference with zero-day samples.

**Table 10:** The relative mRNA expression level of urinary VEGF (u VEGF) at the different time intervals for the control and treated subgroups.

Groups	Zero-day	Before treatment		After treatment	
		1st week	2nd week	1st week	2nd week
G1	1 ± 0.0b	10.6 ± 0.7a	11.5 ± 1.38a	12.6 ± 1.19aA	13.1 ± 1.1aA
G2	1 ± 0.0 d	10.6 ± 0.3 a	11.2 ± 0.45a	9.1 ± 0.13bB	5.4 ± 0.6cB
G3	1 ± 0.0d	12.18 ± 0.7a	9.8 ± 0.13b	7.3 ± 0.74cB	0.77 ± 0.1dC
G4	1 ± 0.0d	11.6 ± 0.5a	11.1 ± 0.05a	8.7 ± 0.61bB	4.1 ± 0.4cB

^a,b^values are significantly different at *P* < 0.05 compared with the control and experimental group with the time in the same row, while ^A,B^ value is significantly different at *P* < 0.05 between groups at the same time point in the same column. Data represented as mean value ± standard error (S.E.) where (n = 5/group). G1: Control positive group; G2: PRP and systemic antibiotic; G3: PRP; G4: Systemic antibiotic.

**Table 11:** The relative mRNA expression level of urinary NGF (u NGF) at the different time intervals for the control and treated subgroups.

Groups	Zero-day	Before treatment		After treatment	
		1st week	2nd week	1st week	2nd week
G1	1 ± 0.0d	7.38 ± 0.6c	8 ± 1.2c	13.3 ± 1bA	26.1 ± 2.3aA
G2	1 ± 0.0c	9.13 ± 0.8a	9.1 ± 0.9a	8.5 ± 0.3aB	5.1 ± 0.1bB
G3	1 ± 0.0c	8.1 ± 0.8a	7.14 ± 1.1a	3 ± 0.2bC	0.69 ± 0.1cC
G4	1 ± 0.0c	9.17 ± 0.9a	10.4 ± 1.3a	9.1 ± 0.3aB	5.6 ± 1.5bB

^a,b^values are significantly different at *P* < 0.05 compared with the control and experimental group with the time in the same row, while ^A,B^value is significantly different at *P* < 0.05 between groups at the same time point in the same column. Data represented as mean value ± standard error (S.E.) where (n = 5/group). G1: Control positive group; G2: PRP and systemic antibiotic; G3: PRP; G4: Systemic antibiotic.

### Assessment of matrix metalloproteinase (MMP-2, MMP-9) activity

**Figure 3:**
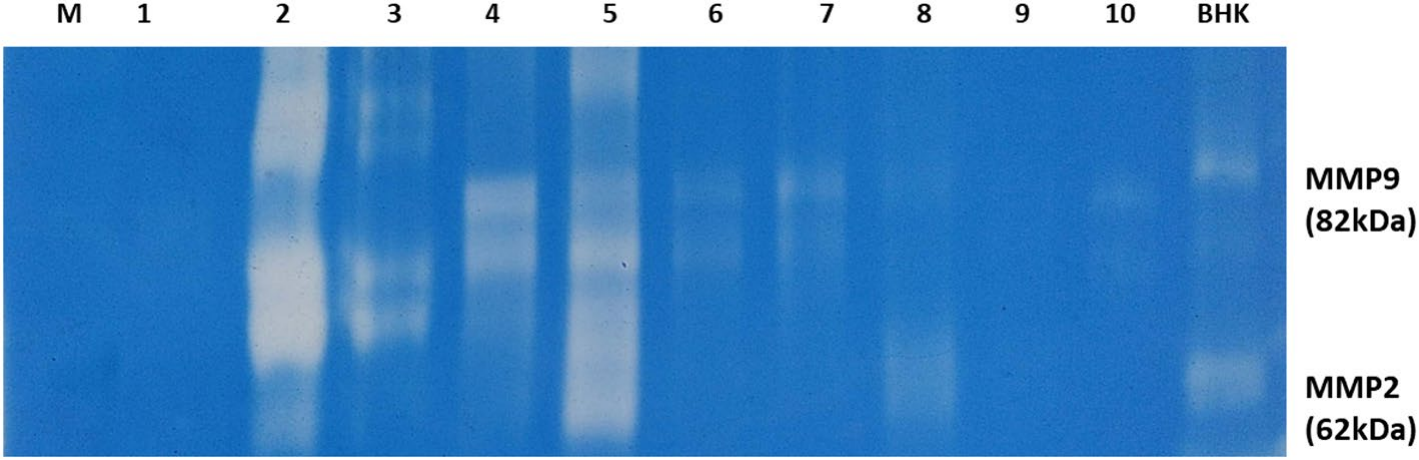
Gelatin zymography for MMPs activity detection in urine (cropped gel) . Lane 1: represent represents zero-day (baseline) samples; Lane 2: 1st-week samples post infection; Lane 3: 2nd-week samples post infection; Lane 4: G2 (PRP and systemic antibiotic) samples at 1st-week post-treatment; Lane 5: G4 (systemic antibiotic) samples at 1st-week post-treatment; lane 6: G3 (PRP) samples at 1st-week post-treatment; lane 7: G2 (PRP and systemic antibiotic) samples at 2nd-week post-treatment; lane 8: G4 ( systemic antibiotic) samples at 2nd-week post-treatment; lanes 9 and 10: G3 (PRP) samples at 2nd-week post-treatment; M: pre-stained protein marker (20-118kDa); BHK lane: is a control marker from baby hamster kidney cells transfected with active MMP-9 (82 kDa) and MMP-2 (62 kDa).

**Figure 4:**
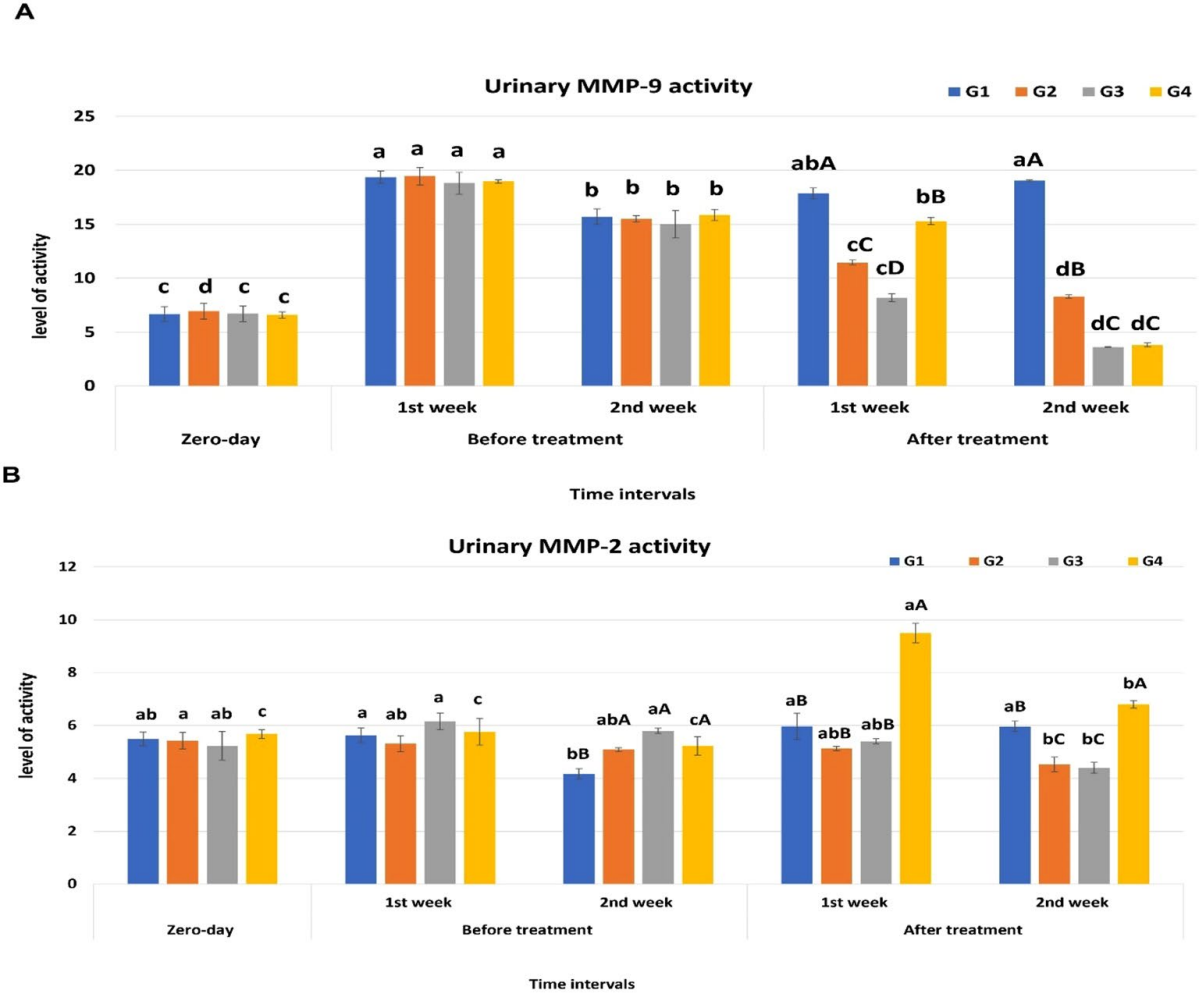
Graphical representation of the changes in the activity of urinary MMPs at different time intervals. (**A**) Representing the activity of urinary MMP-9. (**B**) Representing the activity of urinary MMP-2. ^a,b^values are significantly different at *P* < 0.05 compared with the zero-day samples for the same group at different time intervals, while ^A,B^value is significantly different at *P* < 0.05 between groups at the same time point. Data represented as mean value ± standard error (S.E.) where (n = 5/ group). G1: Control positive group; G2: PRP and systemic antibiotic; G3: PRP; G4: Systemic antibiotic.

Gelatinase activities (MMP-2 and MMP-9) were detected by gelatin zymography Fig. 3. The activity percentage of MMP-9 recorded the highest significant increase at the 1st-week post-infection in all groups in comparison to the zero-day samples, meanwhile; its activity tended to decrease from the 2nd-week post-infection till the end of the experimental period for all groups except for the control positive group. The least MMP9 activity was observed for the G3 (PRP treated group; 3.6 ± 0.04) and G4 (systemic antibiotic group; 3.8 ± 0.17) after the 2nd week of treatment (*p* < 0.05) Fig. 4A. Regarding MMP2 activity, no significant difference was noticed for G1 along the experimental periods (*p* > 0.05). The activity of MMP 2 for G2 and G3 in the 2nd week post-treatment was significantly lowered in comparison to zero-day samples while for G4, the activity was significantly higher at the 1st and 2nd week post-treatment in comparison to the zero-day samples, and the infection periods (*P* < 0.05). Considering the effect of the treatment used; G2 and G3 showed significantly lower activity for MMP2 (4.53 ± 0.27, 4.4 ± 0.2); respectively while G4 recorded the highest activity between groups (6.8 ± 0.14) (*p* < 0.05) Fig. 4B

### Macroscopic evaluation

**Figure 5:**
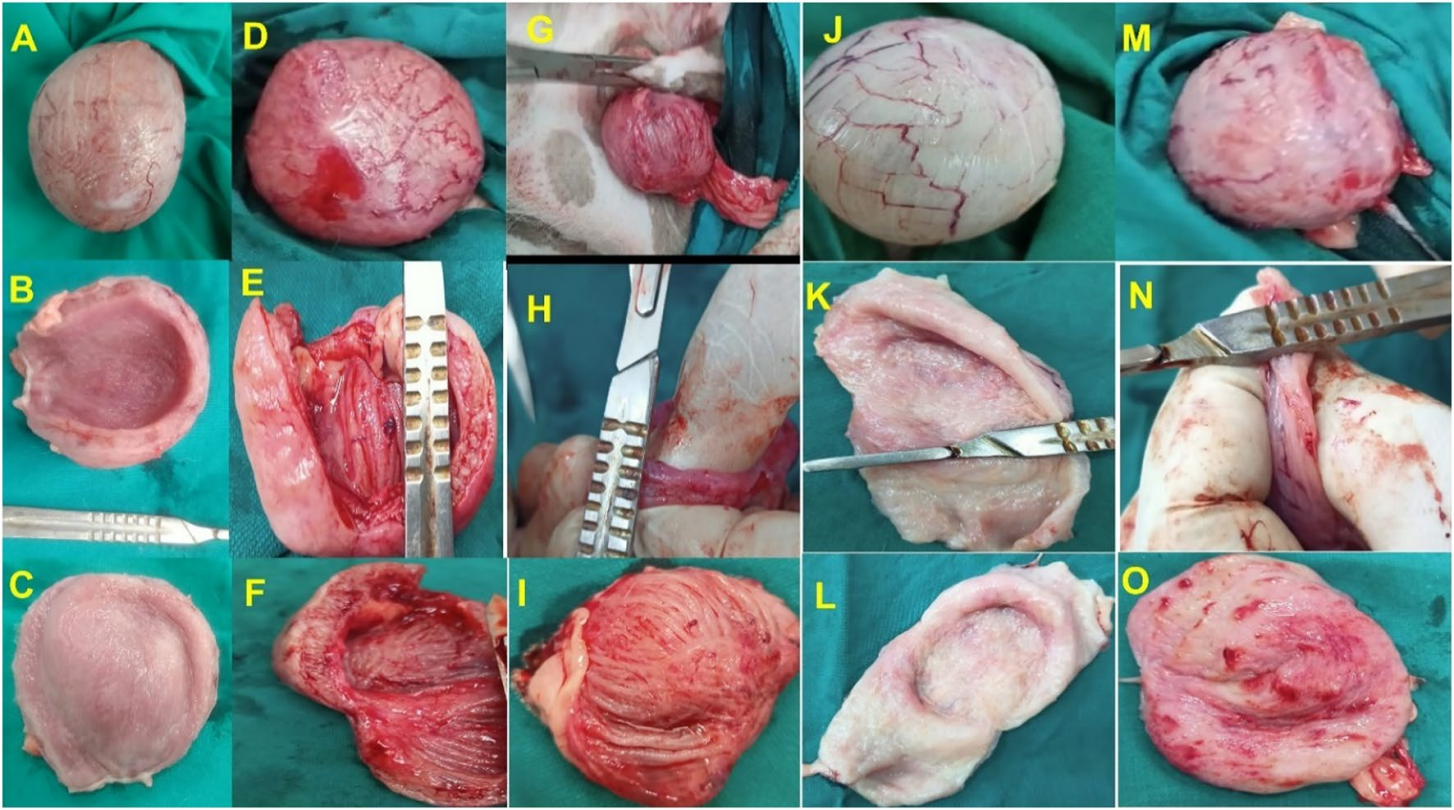
Representative images of the macroscopic observation of the urinary bladder. (**A**–**C**) represent the urinary bladder for the control negative sample; (**D**–**F**) represent the urinary bladder for G1 (control positive) group at the end of the experiment; (**G**–**I**) represent the urinary bladder for G2 ( PRP and systemic antibiotic ) group after, 2nd week post-treatment; (**J**–**L**) represent the urinary bladder for G3( PRP) group, 2nd-week post-treatment, and (**M**–**O**) represent the urinary bladder for G4 (systemic antibiotic) group.

Macroscopic evaluation of the urinary bladder is illustrated in Fig. 5. The urinary bladder wall was normal at the control negative specimen (Fig. 5A) and PRP-treated group (G3) (Fig. 5J), moderate congestion was observed in the systemic antibiotic-treated group (G4)( Fig. 5M), while severe congestion was observed in the control positive group (G1 ) ( Fig. 5D ) and PRP with a systemic antibiotic-treated group (G 2 )( Fig. 5G). The urinary bladder wall thickness was normal in the control negative specimen (Fig. 5B) and PRP-treated group (G3) (Fig. 5K), while it is mildly thickened in PRP with a systemic antibiotic-treated group (G 2) (Fig. 5H) and systemic antibiotic-treated group (G4) (Fig. 5N) and severe thickened in G1 (Fig. 5E). The bladder luminal wall was normal in the control negative specimen (Fig. 5C) and G3 (Fig. 5L), mild congested in G2 (Fig. 5I), moderate congestion with severe erosions in G4 (Fig. 5O) and severe congestion in G1 (Fig. 5F).

### Histopathological findings

**Figure 6:**
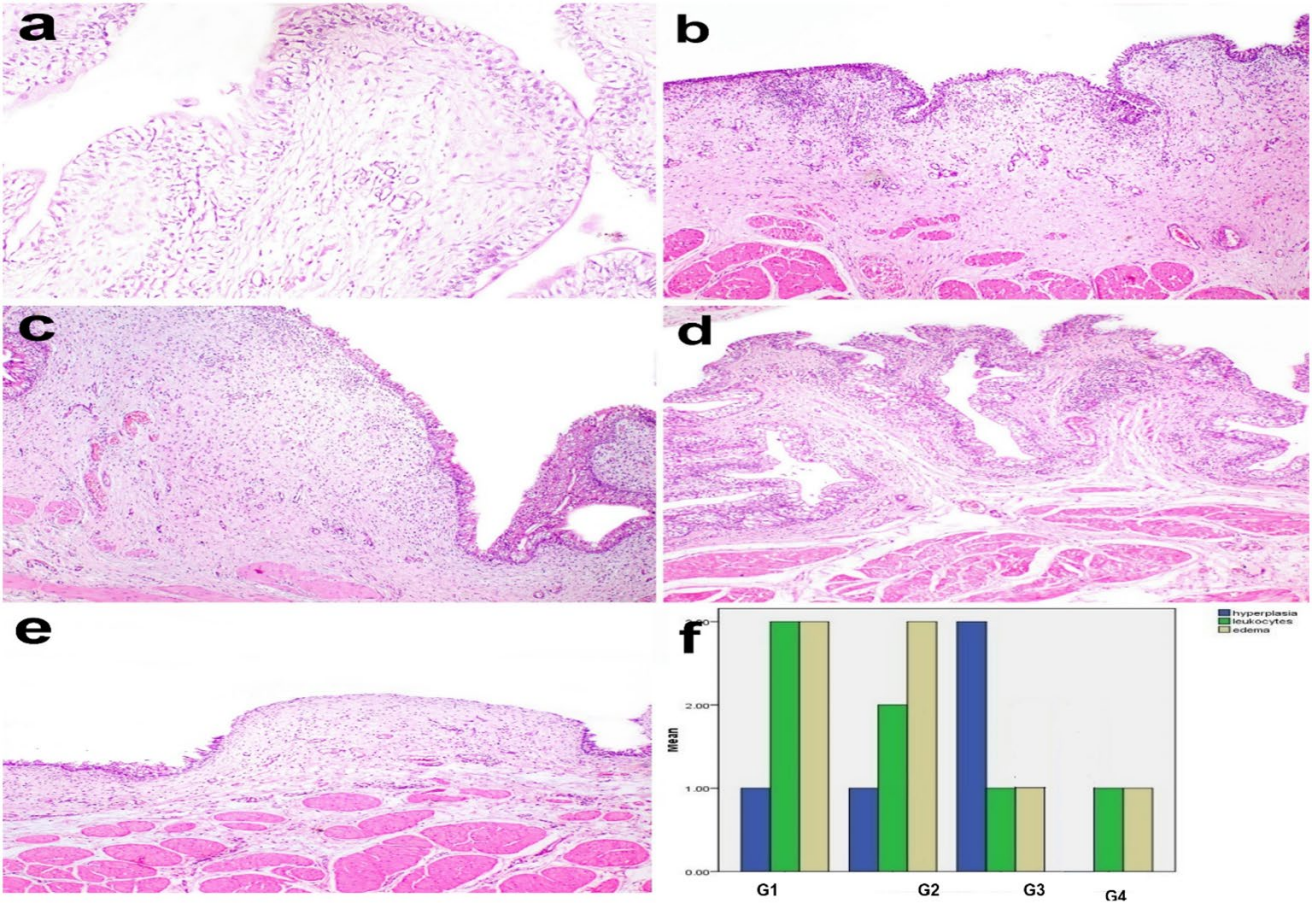
Histopathology of the urinary bladder in dogs. (**a**) normal histological structure of the urinary bladder in control negative samples (**b**) erosions in the urothelium, congestion of blood vessels, severe edema, and severe diffuse leukocyte infiltration in the lamina propria are observed in G1. (**c**) mild hyperplastic urothelium and moderate leukocyte infiltration with severe edema in lamina propria are observed in G2. (**d**) moderate urothelium hyperplasia, mild leukocyte infiltration, and mild edema in lamina propria are observed in G3. (**e**) focally eroded urothelium and mild leukocyte infiltration with moderate edema in lamina propria are observed in G4. Hematoxylin and eosin stain X100. (**f**) Bar chart of lesion scores in different groups. G1: Control positive group; G2: PRP and systemic antibiotic; G3: PRP; G4: Systemic antibiotic.

Microscopy of the urinary bladder in the control negative samples at zero-day revealed normal histological structure (Fig. 6a). In the control positive (G1) group, microscopy of the urinary bladder revealed erosions in the urothelium, congestion of blood vessels, severe edema, and severe diffuse leukocyte infiltration in the lamina propria (Fig. 6b). In G2, the urothelium was moderately hyperplastic, and lamina propria was moderately infiltrated with leukocytes and thickened by severe edema (Fig. 6c). In G3, the urothelium was intact with no evidence of hyperplasia and the lamina propria was mildly edematous and mildly infiltrated with leukocytes (Fig. 6d). In G4, the urothelium was focally eroded and lamina propria was moderately edematous and mildly infiltrated with leukocytes (Fig. 6e).

The score of leukocyte infiltration was significantly decreased in G3 compared to G1. All parameters evaluated were significantly altered in G3 in which the edema and leukocyte infiltration were significantly decreased compared to G1 whereas the urothelium hyperplasia was significantly increased compared to G2, and G4 (Fig. 6f). .

### Immunohistochemistry

**Figure 7:**
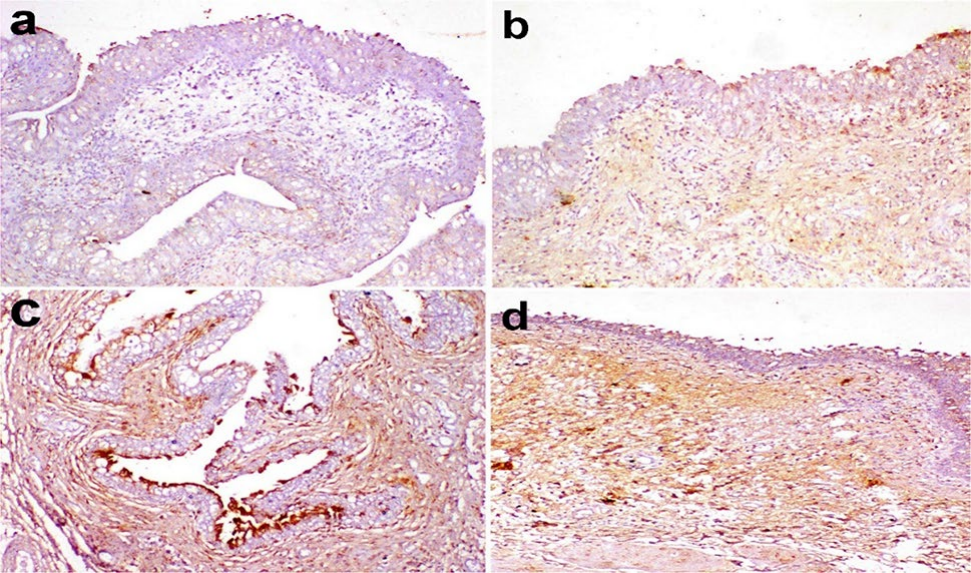
Immunohistochemistry of Cox-2 expression in the urinary bladder of dogs. (**a**) mild expression in the urothelium and moderate expression in inflammatory cells infiltrating the lamina propria in G1. (**b**) moderate expression in the urothelium and inflammatory cells in G2. (**c**) severe expression at the apical cells of the urothelium and moderate expression in the inflammatory cells. in G3. (**d**) moderate expression in the urothelium and severe expression in the inflammatory cells in G4. Immunoperoxidase and hematoxylin counterstain X 200. G1: Control positive group; G2: PRP and systemic antibiotic; G3: PRP; G4: Systemic antibiotic.

In G1, cox-2 was expressed mildly in urothelium however was expressed in inflammatory cells infiltrating the lamina propria. In G2, moderate Cox-2 expression was observed in the urothelium and inflammatory cells. In G3, cox-2 was severely expressed in the apical cells of the urothelium and moderately in the inflammatory cells. In G4, cox-2 was moderately expressed in the urothelium and severely expressed in the inflammatory cells infiltrating the lamina propria (Fig. 7).

**Figure 8:**
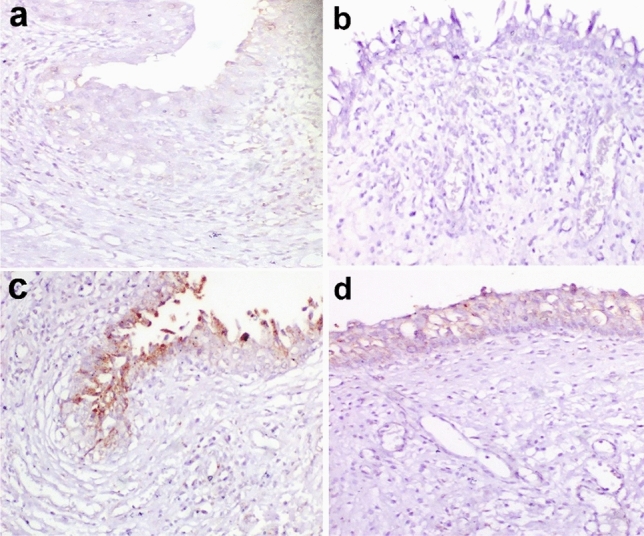
Immunohistochemistry of NF-κβ P65 expression in the urinary bladder of dogs. (**a**) mild nuclear translocation of NF-κβ in inflammatory cells in G1. (**b**) diminished expression of NF-κβ expression in G2. (**c**) severe NF-KB expression in the apical cells of the urothelium in G3 (**d**) mild nuclear expression of NF-KB in urothelial cells in G4. Immunoperoxidase and hematoxylin counterstain X 400. G1: Control positive group; G2: PRP and systemic antibiotic; G3: PRP; G4: Systemic antibiotic.

The activated form of NF-κB P65 was observed in the nucleus of inflammatory cells in G1 however, its expression was almost diminished in G2. In G3, severe NF-κB P65 expression was observed in the apical cells of the urothelium. In G4, urothelial cells only showed mild nuclear expression of NF-κB P65 (Fig. 8).

**Figure 9:**
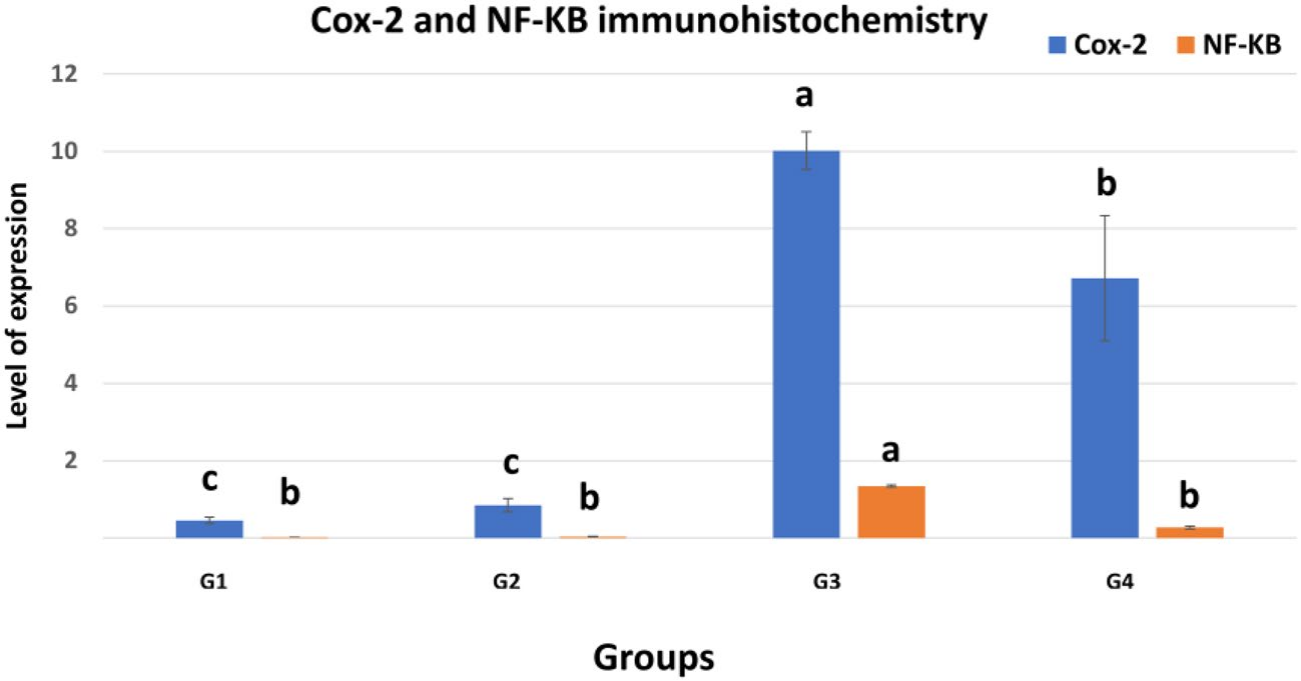
Immunohistochemical analysis for area% of Cox-2 and NF-κβ P65 in examined urinary bladders at the end of the experiment. Data are expressed as the mean ± SEM for triplicate samples (n = 5). Bars bearing different lowercase letters (**a**–**c**) are significant at *P* < 0.05. G1: Control positive group; G2: PRP and systemic antibiotic; G3: PRP; G4: Systemic antibiotic.

The area percent of cox-2 expression in the urinary bladder was significantly elevated in G3 and G4 compared to G1 and G2 (Fig. 9). On the other hand, the area percent of NF-κB P65 was significantly elevated only in G3 (Fig. 9).

## Discussion

The bladder epithelium is commonly invaded by UPEC despite having robust barriers and antimicrobial bladder epithelial cells and is the most targeted site for UTIs^57^. In the current investigation, our goal was to assess the effectiveness of platelet-rich plasma (PRP) as a substitute, regenerative treatment for bacterial cystitis.

### B-mode and Doppler ultrasound assessment

After receiving PRP treatment, dogs with acute cystitis showed less bladder thickness as measured by B-mode. In many pathological conditions, the increased bladder thickness have been recorded using B-mode sonsography^58,59^. The usefulness of using PRP in the treatment of lower urinary tract disorders has been reported in many studies and this effect was attributed to its ability to resolve the bladder inflammation and regeneration of bladder epithelium^13,60–63^.

In the present study, pulsed-wave Doppler demonstrated an increased pattern in PI, which was linked to a decrease in the urethral artery's PV following treatment. The control animals had lower PV than the animals experiencing acute cystitis. There are two possible causes for the elevated urethral PI: a decrease in arterial velocity^64–66^ or a significant increase in blood peak and end velocities^44,45^. Intravesical PRP instillation was reported to prevent uropathogenic bacteria from destroying epithelium and disrupting urothelial homeostasis, which in turn improves urothelial barrier function in patients suffering from bladder infections. PRP treatment is thus based on cell proliferation and migration to the injured region, followed by cell differentiation and angiogenesis^63,67,68^.

### Microbiological evaluation

The aggressive reactions that bladder epithelial cells (BECs) adopt in response to UPEC result in significant neutrophil recruitment, BEC mortality, and exfoliation, all of which help to transport a lot of UPEC into the urine^69–71^. In the current study, UPEC was found in urine at significantly higher levels during the infection period, while during treatment, the combination of PRP and the antibiotic showed their superiority in the elimination of infection. PRP exhibited antimicrobial qualities in rats' experimentally infected surgical wounds^72^, osteomyelitis models in rabbits^73,74^, post-operative spinal implant-associated infections^75^, canine skin wounds infected with MRSA^39^, and enhanced the healing of infected wounds in the diabetes rat model^76^. PRP produces hydrogen peroxide, which is responsible for its antimicrobial activity. This produces a ROS medium that is inhospitable to microorganisms^77^.

PRP and urinary oxidant/ anti-oxidant status: Blood indices can't be used to diagnose lower urinary tract infections since systemic infection usually does not occur in cystitis^2^. During UTI many biochemical changes occur and can appear in the urine; hence, urine can be considered a mirror for the inflammatory environment of the bladder and urinary system^78,79^. The current state of an individual's lower urinary tract functions can be reflected through the profiling of urinary oxidative and antioxidative stress biomarkers^80^ which have diagnostic as well as prognostic values^7^.

One biomarker used to assess the antioxidant potential of bodily fluids, including urine, is the total antioxidant capacity (TAC)^81–83^. Urinary TAC in the current study decreased during infection episodes and tended to increase as various treatments were initiated. However, the levels of TAC did not increase like at the beginning of the study but both PRP and PRP with antibiotics showed their superiority in the improvement of TAC to antibiotic solely. Endogenous oxidative stress was linked to UPEC growth in the urine^84^. TAC was found to be lower in the urine of patients with renal diseases than in the urine of healthy individuals, according to^79^. Urinary antioxidant enzymes were damaged by *E. Coli* UTIs in diabetic patients, which led to oxidative stress and a decrease in urinary TAC^85^. PRP has been found to have anti-apoptotic and antioxidant properties in mammalian cells^86^. Before cryopreservation of semen; PRP is added to the semen extender to increase the antioxidant status and reduce the oxidative stress^87–90^. PRP was reported to improve ovarian TAC levels and diminish MDA levels in PCOS-induced pathogenesis^91^.

One of the most significant manifestations of oxidative stress brought on by ROS is lipid peroxidation (LPO). Urinary Malondialdehyde (MDA) and its derivatives have been used as indicative for several diseases^92–94^. In UTIs, MDA reports can be obtained before the results of cultures and it may be used as an early diagnostic tool to start treatment without waiting for the results of the cultures^27^. Our results showed the capability of the different treatments to reduce the urinary MDA levels after its elevation during the infection. MDA levels were reported to increase in pregnant women with UTIs^95^ during acute and chronic cystitis in camels^96^, and in diabetic patients with *E. coli* UTI^85^. In rats exposed to d-gal-induced testicular oxidative stress^97^. PRP intratesticular injection can lower MDA levels^98^. Dog wounds treated with PRP had lower MDA levels^99^. Combining PRP and stromal vascular fraction cells (SVFs) reduced MDA levels in the deep dermal burn injury model^100^.

Nitrite (NO2-) is one of the metabolic end products derived from the oxidation of NO and excreted in urine^101^. If excess nitrite is generated, either by the cells or due to local inflammation; it can be reduced back to NO ; nonenzymatically^102^ or by bacterial enzymes^103^. Since NO and its metabolites are known to inhibit the growth of a wide variety of bacterial species, including *E. coli*, NO may contribute to the bladder defense mechanisms during bacterial infection^104,105^. In the present study, urinary nitrite was elevated during the infection period and remained higher than the zero-day samples even after treatment. In UPEC-induced UTI animal models, elevated levels of urine nitrite and iNOS have been documented^106–108^. Our histological and immunohistochemical results, along with the previous reports demonstrate that uroepithelial cells expressed iNOS later in the UPEC infection and for a longer duration than neutrophils, all contribute to the explanation of our results^108^.

### PRP and matrix metalloproteinases

Gelatinases (MMP-2 and MMP-9) change basal lamina molecules during various cellular processes, such as angiogenesis and neurogenesis, which ultimately results in cell death^109–111^. Recently, MMP9 was reported to be one of the promising biomarkers for UTIs which needs further studies^112^. So, we aimed to follow up on its activity during the UTI and the effect of different treatment strategies for the first time in dogs. Our results showed an increase in the MMP9 activity during the infection and a significant reduction after using the different treatments. Meanwhile, for MMP-2 activity didn’t show any change during the infection, but it significantly decreased with PRP and PRP with antibiotic treatment. The most confusing result is the increase of MMP-2 with antibiotic treatment only. As a result, MMPs were differentially regulated during the experimental UTI rather than responding in an all-or-none pattern. In the same line, the differential expression for MMP-2 and MMP-9 was also noticed in experimental meningitis in rat models where MMP-9 was upregulated during the infection and with antibiotic treatment while MMP-2 remained unchanged^113^.

Urinary MMP-9 was found to be elevated in patients with UTIs as neutrophils secrete MMP-9 as a defense mechanism^71,114,115^ and in cystitis^116,117^. MMP-9 and MMP-2 production was totally or partially inhibited following the PRP administration in ankle OA rats^118^and the OA cell model^119^, and in canines as well as cats with corneal ulcers^42^. Our study is contradictory to an in vitro study which showed that MMP-2, -3, and -9 are present in the different PRP preparations, and their release over a minimum of six days^120^. Due to the notable increase in MMP-2 activity following antibiotic treatment and our macroscopical analysis, the erosions seen in the bladder may be caused by the gelatinase activity of MMP-2. This explained by the possibility that antibiotics, in addition to their antimicrobial effects, also indirectly modulate the immune system^121^. Therefore, Liu et al., 2008^113^ previously suggested the beneficial effect using of dexamethasone in parallel with antibiotic treatment in experimental meningitis to overcome the deleterious effect of MMPs. Furthermore, some research revealed that MMP-2 and MMP-9 could be secreted in vitro by both smooth muscle cells and urothelial cells^122^.

### PRP and growth factors related genes

PDGF is one of the numerous growth factors regulating the cell growth and division and crucial for the development of blood vessel formation and mesenchymal cell differentiation^13^. In the present study, PDGF-B was significantly elevated in all groups except the control positive one in which it downregulated and the marked increase was for the PRP-treated group. In a clinical trial, PDGF BB was used to treat patients with chronic pressure ulcers^123^. Chitosan-based antibacterial nanofibers loaded with VEGF and PDGF-BB were used in vitro and in rat skin wound models to promote angiogenesis as well as remodeling, respectively. PDGF-BB was found to help in epithelium regeneration, tissue remodeling, and accelerate wound healing^124^.

Specific target neurons are stimulated to differentiate and survive by a small protein called nerve growth factor (NGF), which is secreted by the smooth muscle and urothelium^125^. NGF may play a part in the diagnosis and treatment of lower urinary tract dysfunction as a urine biomarker^126–128^. Neuronal hypersensitivity and alterations in the function of the urinary bladder were linked to the overexpression of NGF in the bladder tissue and urine^126,129–132^. Inflammation of the bladder mucosa and underlying nerves during the acute stage of UTI, results in uncomfortable and irritative signs^133,134^. In the same line, our clinical examination showed that all groups suffered from polyurea all over the experimental period due to the upregulation of u NGF except for PRP treated group which was downregulated. In line with our findings, Chuang et al.,^135^ found that u NGF levels rose in women with acute UTI. NGF is associated with both acute and chronic inflammation in the bladder wall following acute UTI. Additionally, after receiving antibiotics, the level of urine NGF decreased, but at 12 weeks following the UTI episode, it was still higher than the control^135^. Furthermore, u NGF was increased in IC/BPS patients and decreased following repeated intravesicular PRP instillations^13^.

A master regulator of either pathological or physiological angiogenesis is VEGF^136^. It has been reported to occur during viral and bacterial infections^137,138^. Variations in VEGF levels are linked to modifications in the urinary bladder's vascular structure^139^. since infiltrating lymphocytes and other inflammatory cells may represent additional sources of VEGF besides the bladder source as a survival action, VEGF can increase vascular permeability, leading to glomerulations, edema, and the establishment of a progression of disease pathophysiology^136^. Installation of VEGF was found to increase the sensory nerve density through the urothelium and alter bladder function as well as visceral sensitivity. it has been suggested that axons may direct blood vessels and vice-versa via VEGF^140–142^ and signals from vessels, like the neurotrophins NGF are required for orchestrating the extension of neurons adjacent to vessels^143,144^. Therefore, using neutralizing VEGF antibodies greatly lessens inflammation in the bladder^145^. Intravesical instillation of PRP was able to protect rabbit bladders from hemorrhagic cystitis after treatment with hydrochloride^146^. In IC/BPS patients uVEGF was reduced by the third PRP intravesicular injection^13^. Intravesical PRP instillation has been shown to reduce bladder bleeding in a rat model of hemorrhage induced by cyclophosphamide^147^.

Altogether, this can explain our findings concerned with the parallel upregulation of VEGF and NGF in all groups except for PRP treated group which was downregulated. Also, can explain our macroscopical findings in which the edema and congestion were resolved in the PRP-treated group other than in the remaining groups.

### Histopathological evaluation

After receiving antibiotic therapy for bacterial cystitis, pathogenic *E. coli* will produce intracellular bacterial communities (IBC) in the urothelial cells^148,149^. So, it is crucial to enhance urothelial regeneration and differentiation to eradicate the germs that are concealed in dysfunctional urothelial cells^60^. Although the bladder mucosal urothelial cells divide infrequently or not at all under normal circumstances, any urothelial damage caused by chemicals, toxins, or bacterial infections prompts fast proliferation^150^.

In the current study, all treatments evaluated improved the histopathological findings in all groups compared to the control positive one. However, PRP treated group was remarkably improved in which the normal thickness of urothelium was maintained and edema was regressed. Similarly, An earlier investigation revealed that PRP had a positive effect on ketamine-induced cystitis in rats in which the urothelium thickness was increased but congestion and inflammatory cell infiltration were still observed in the urinary bladder^151^. In the present study, all groups treated with PRP showed urothelium proliferation which is critical in maintaining a functional barrier against urinary toxic substances and bacteria^60^. Therefore, it is suggested that PRP improves the regenerative capacity of the urothelium. The decreased submucosal thickness due to regression of edema in treated groups was similarly reported in a previous study on cyclophosphamide-induced acute interstitial cystitis treated with PRP in rats^152^. Meanwhile, in a study by Ozyuvali et al.,^147^; the histological changes were not significant in single PRP-treated rat bladders with cyclophosphamide-induced hemorrhagic cystitis which suggests that the histological alterations for successfully treated cystitis models may manifest following several PRP treatments. PRP injections induce the release of growth factors into the suburothelium, which aids in both urothelial regeneration and the repair of damaged urothelium^60^. PRP injections can promote the expression of barrier proteins and increase progenitor cell proliferation^41^.

Although antibiotic treatment reduced the urinary bacterial count as it was toxic for the microorganism, it was toxic to the urotheliuminhibited the tissue hyperplasia, and resulted in tissue erosions. Meanwhile, these erosions were not observed in the PRP and antibiotic-treated group due to the ameliorative effect of the PRP. Hence, PRP can be an alternative safe regenerative therapy for UTI.

### Immunohistochemical evaluation

The active form of NF-κB is a crucial player in the process of inflammation^20^. Its expression was mainly observed in the urothelium and the lamina propria of PRP-treated cystitis^151^; likewise to our study which showed expression of NF-KB in the urothelium and inflammatory cells of the PRP-treated groups. However, the decreased expression of NF-κB in the group treated with PRP and antibiotics was not reflected in the histopathology of the bladder mucosa as there were still erosions in the urothelium. This might be due to the conjugation of PRP with antibiotic as the group that was treated with antibiotic only revealed massive areas of ulceration and erosions although the inflammation was regressed.

PRP also increased Cox-2 expression in urothelium in all PRP-treated groups, similarly Cox-2 expression was observed in the urothelium and sub-urothelial layer in ketamine-induced cystitis in rats^21^. Contrary to our findings, better epithelization was observed in rats treated with Cox-2 inhibitors. Most of the literature available indicated that PRP decreased cox-2 expression^153,154^. Cox-2 expression was mainly concentrated in the apical cells of urothelium which might be due to its direct exposure and contact with bacteria present in the urinary tract. This could also be supported by another study which indicated that PRP enhanced the proliferation of chondrocytes but didn’t reduce the inflammation^155^. The fact that PRP secretes various cytokines that can trigger new inflammatory processes and aid in the resolution of previously unresolved inflammation helps to explain these results^156^.

## Conclusion

To the authors’ knowledge, this is the first report in dogs evaluating the efficacy of using intravesicular instillation of autologous PRP for the treatment of UPEC-induced cystitis in comparison to conventional antibiotics. Despite that the current study has several limitations including, using male dogs, hence, sex bias might have existed and the limited number of samples, we were able to find the potential of PRP as antimicrobial and regenerative therapy that needs to be confirmed in future clinical studies. PRP has improved the redox status of the animals and the VEGF-induced neurogenic inflammation and reduced the MMPs activity. Conventional antibiotic in the present study did not show marked improvement except for their antibacterial effect and it showed a mild hindering effect for the action of PRP when they are used in combination. Using PRP alone in the present study showed marked clinical improvement of diseased animals, which results in a better quality of life and animal welfare. Further investigations are needed to figure out the mechanism of the anti-inflammatory regenerative properties of the PRP.

### Supplementary Information

Supplementary Information.

## Data Availability

Data is provided within the manuscript or supplementary information.

## Abbreviations

COX-2: Cyclooxygenase-2
IC/BPS: Interstitial cystitis/bladder pain syndrome
MDA: Malondialdehyde
MMPs: Matrix metalloproteinases
NGF: Nerve growth factor
PDGF: Platelet-derived growth factor
TAC: Total antioxidant capacity
UPEC: Uropathogenic *Escherichia coli*
VEGF: Vascular endothelial growth factor
