# Supplementary material for: Platelet-rich plasma attenuates the UPEC-induced cystitis via inhibiting MMP-2,9 activities and downregulation of NGF and VEGF in Canis Lupus Familiaris model
Source: Sci Rep. 2024 Jun 13;14:13612. doi: 10.1038/s41598-024-63760-y (PMC11176177; doi:10.1038/s41598-024-63760-y)
Supplement: Supplementary file 1 — Supplementary Information. [file 41598_2024_63760_MOESM1_ESM.docx]

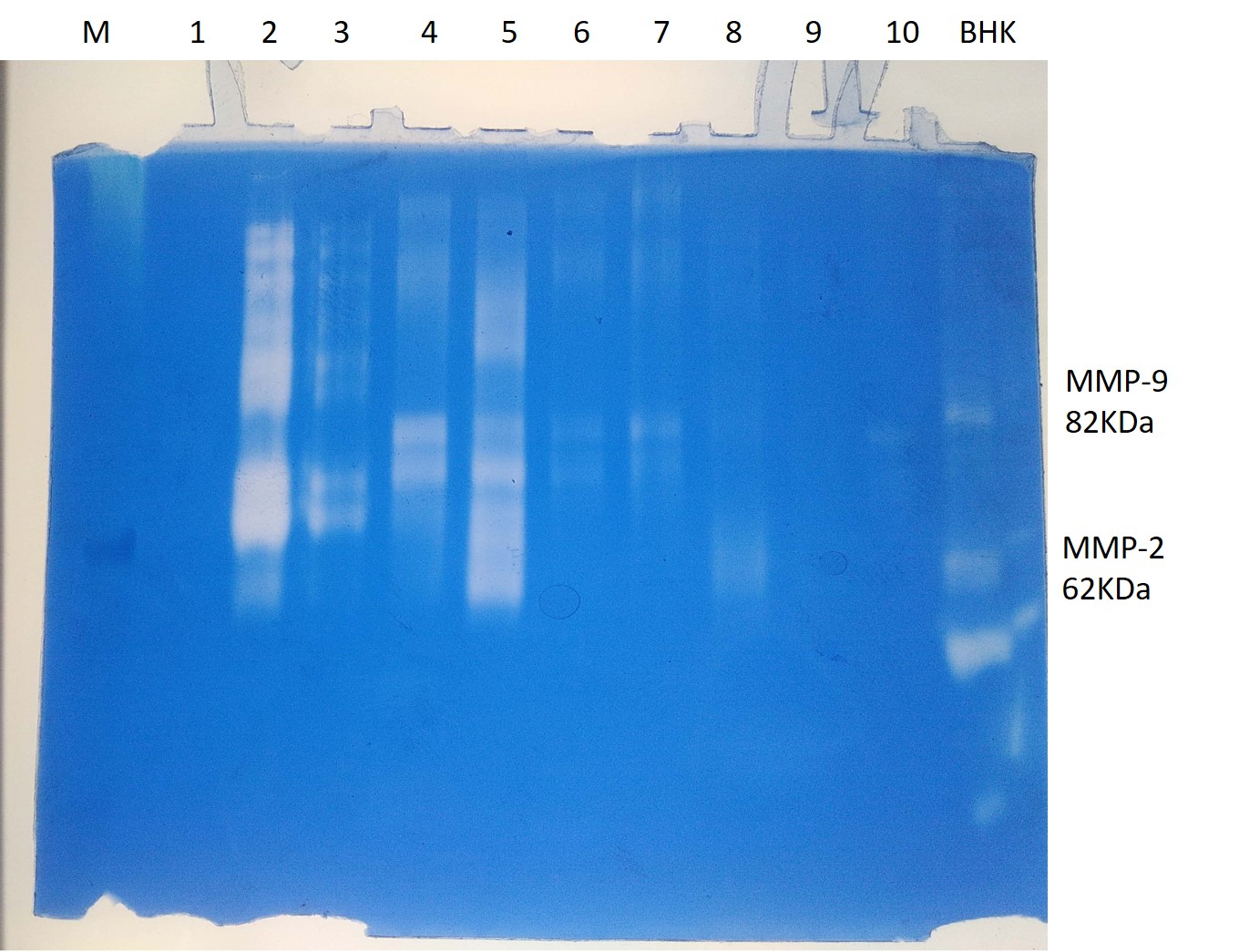


**Figure 3:** Gelatin zymography for MMPs activity detection in urine (uncropped gel) . Lane 1: represent represents zero-day (baseline) samples; Lane 2: 1^st^-week samples post infection; Lane 3: 2^nd^-week samples post infection; Lane 4: G2 (PRP and SYS.) samples at 1^st^-week post-treatment; Lane 5: G4 ( systemic antibiotic) samples at 1^st^-week post-treatment; lane 6: G3 (PRP) samples at 1^st^-week post-treatment; lane 7: G2 (PRP and SYS.) samples at 2^nd^-week post-treatment; lane 8: G4 ( systemic antibiotic) samples at 2^nd^-week post-treatment; lanes 9 and 10: G3 (PRP) samples at 2^nd^-week post-treatment; M: pre-stained protein marker (20-118kDa); BHK lane: is a control marker from baby hamster kidney cells transfected with active MMP-9 (82 kDa) and MMP-2 (62 kDa).


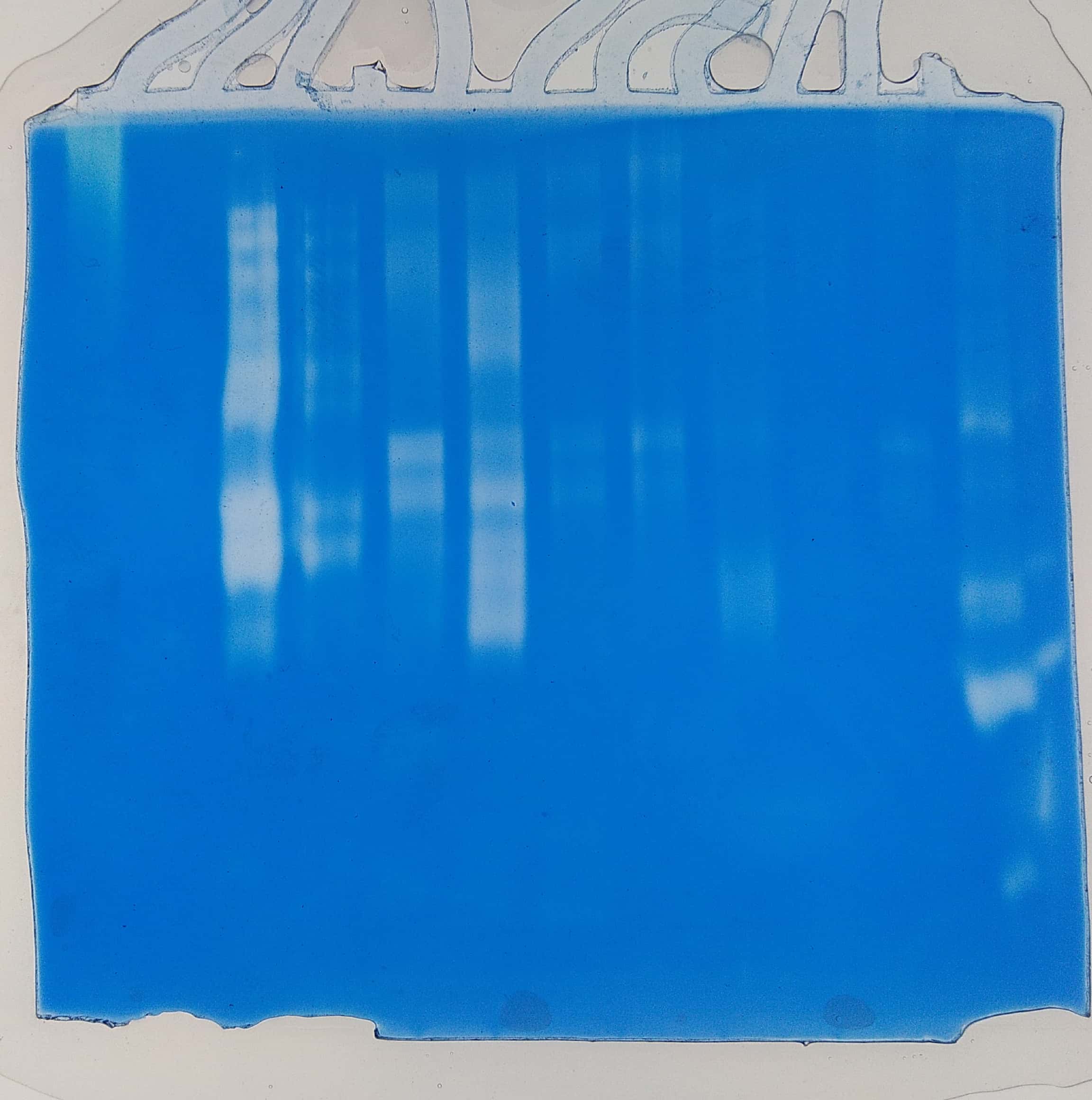


**Figure 3:** Gelatin zymography for MMPs activity detection in urine (uncropped gel)
